# Measurement of the scalar curvature of high-power lasers

**DOI:** 10.1038/s41598-022-23045-8

**Published:** 2022-10-27

**Authors:** Antonela Toma, Octavian Postavaru

**Affiliations:** Center for Research and Training in Innovative Techniques of Applied Mathematics in Engineering, University Politehnica of Bucharest, Splaiul Independentei 313, 060042 Bucharest, Romania

**Keywords:** Mathematics and computing, Physics

## Abstract

High-power lasers develop high energy per unit time, and as energy curves space, we expect atomic energy levels to change. The fluorescence spectrum is a good measurement of the matrix elements involved in the Rabi oscillation and consequently allows us to determine the scalar curvature. At high *Z*, electrons oppose ionization even for strong intensities. Because high-power lasers address relativistic atoms, the wave functions involved must be solutions of the Dirac equation in a curved space-time. The paper can be seen as a way to check whether the Einstein’s gravitational theory is valid in the dimension of laboratory.

## Introduction

With the advent of lasers in the X-ray domain, there is a growing interest in expanding laser spectroscopy in this field. Optical light spectroscopy allowed us to measure magnetic dipoles and multipoles with an accuracy that allowed us to understand the effects of QED, such as the electron anomalous magnetic moment^1^. Using collinear laser spectroscopy, isotope shifts in different atomic species were measured, which allowed the analysis of the collective structure of the nuclei^2^. In addition to nuclear effects, the interaction of the correlated motion of electrons with that of the nucleus can be studied with the help of the trapped-ion method^3^.

The rapid progress in the development of free-electron laser facilities allows us to address ultra-short pulses of high intensities^4^. A novel technique for absolute wavelength determination in high-precision crystal X-ray spectroscopy recently introduced has been upgraded reaching unprecedented accuracies^5^. In order to determine the Bragg angles, the Bond method is applied to visible laser rays. In the work^6^, in order to verify the oscillator strength of Fe$^{16+}$, an experiment was employed in order to analyze the fluorescence of an iron target that interacts with the X-rays emitted by a free-electron laser. The paper^7^ used a new technique for the production and storage of radioactive ions to measure nuclear effects in the X-ray transitions of few-electron heavy ions. Variations of the fine-structure constant $\alpha$ can be obtained by comparing the spectra of highly charged ions with those of light ions^8^. Also, to test fundamental physics, the paper^9^ showed that hole transitions in multiply charged ions are extremely high sensitivity to $\alpha$ variation.

The determination of matrix elements and frequencies by measuring the distance between the sidebands of the Mollow spectrum was highlighted both in^10^ where the author studied a two-level configuration interacting with a short-wavelength laser field, as well as in^11^ where the authors analyzed a configuration of three energy levels under the simultaneous action of a laser x and an optical one. The last work was successfully extended to He-like systems^12^. In general, energy curves space and we expect high-power lasers to have the same effect. Applying the above-mentioned techniques, we can extract matrix elements by measuring the distance between the sidebands of the fluorescence spectrum at resonance, and we can determine the curvature.

The theoretical description of fermions in curved space-time can be found for example in^13^. In the work^14^, for a space-time of Melvin type the authors obtained a relativistic wave equation for spin 1/2 particles. In this space the metric is determined by a magnetic field, allowing the study of very strong magnetic fields on energy levels, phenomena existing in magnetars or in ultra-relativistic collisions. The covariant Dirac equation in a $2+1$ dimensional space-time in the presence of an electromagnetic field is studied in^15^. Using a unitary transformation in a polar coordinate system, it is shown that the Dirac equation can be transformed into a Schrödinger type differential equation for one of the spinor components. The Dirac equation in a curved space was also applied in condensed state physics to study massless particles in graphene^16^. Using ultracold atoms connected by an optical network, in the work^17^ a Dirac field was simulated near an event horizon. Such a quantum simulator allows the observation of the Unruh effect. It is important to emphasize that the particles in field theory are observer-dependent^18^, and consequently, this fact must be taken into account when we study the emission of particles from black holes or cosmological horizons. In the work^19^, the propagation of fermions in a curved space-time created by an artificial gravitational field of cold atoms was simulated.

In literature, experiments are known to test space-time curvature with the help of atomic interferometry, which measures the wave characteristic of atoms to determine tiny differences in phase as the atoms traverse the arms of an interferometer. The Aharonov–Bohm effect is a quantum effect that highlights how a magnetic field affects the phase of an electron wave propagating along a wire.

In the paper^20^, the authors measured the gravitational phase shift induced in a matter-wave interferometer by a kilogram-scale source mass close to one of the wave packets. The results show that the phase shift due to the interaction of atoms with a large mass is consistent with the Aharonov–Bohm effect. The paper^21^, presented a single-source dual atom interferometer and utilize it as a gradiometer for precise gravitational measurements. Due to gravitational curvature effects due to a Pb source, the gradiometer measures a phase shift of 1 rad.

In this paper we use the relativistic fluorescence spectrum to determine the Rabi frequency corresponding to the oscillation in an ion with two energy levels in a high intensity X-ray field. The wave functions used are solutions of the Dirac equation in a curved space-time. This combination of quantum mechanics and gravity allows us to investigate the connection between laser intensity and laser-induced scale curvature in the atom.

The present paper has the following structure: in the section “Theory” we present the fundamental theory of the relativistic fluorescence spectrum and of the wave functions involved, which are solutions of the Dirac equation in curved space-time. In the section “Results”, we present the way we can calculate the scalar curvature in highly charged uranium. A data table corresponding to other highly charged ions is also presented. Throughout the work we use natural units.

## The theory

In the present paper, we analyze the fluorescence spectrum for relativistic atoms in curved space-time. To describe the relativistic spectrum for an ion with two energy levels, we must replace the non-relativistic Rabi frequencies with the relativistic ones^10^. In the presence of a classical monochromatic electromagnetic field, the fluorescence spectrum $S^{(\lambda )}(\omega _f)$ is completely described by the Rabi frequency $\Omega _{(\lambda )}$ together with decay width $\Gamma$ of the upper level^10,22^

1 $$\begin{aligned} S^{(\lambda )}(\omega _f)=\frac{\Gamma }{\pi }\frac{2\Gamma ^2+\left. \Omega _{(\lambda )}\right. ^2+2(\omega _f-\omega _L)^2}{\Gamma ^2+2\left. \Omega _{(\lambda )}\right. ^2 +4\Delta ^2}\frac{4\Gamma \left. \Omega _{(\lambda )}\right. ^4}{X_0+X_2\Gamma ^2+X_4\Gamma ^4+X_6\Gamma ^6}, \end{aligned}$$

where

$$\begin{aligned} X_0= & {} 16\left[ \Delta ^2+\left. \Omega _{(\lambda )}\right. ^2-(\omega _f-\omega _L)^2\right] ^2(\omega _f-\omega _L)^2,\\ X_2= & {} 4\left[ 6(\omega _f-\omega _L)^4-2\left( 3\Delta ^2-\left. \Omega _{(\lambda )}\right. ^2\right) (\omega _f-\omega _L)^2\right. +\left. (2\Delta ^2+\left. \Omega _{(\lambda )}\right. ^2)^2\right] ,\\ X_4= & {} 8\Delta ^2+4\left. \Omega _{(\lambda )}\right. ^2+9(\omega _f-\omega _L)^2,\\ X_6= & {} 1, \end{aligned}$$

and where $\Delta =\omega _{L}-\omega _{12}$. We noted with $\omega _{L}$ the laser frequency and with $\omega _{12}$ the transition frequency between the two energy levels. The $\lambda =\{-,0,+\}$ parameter indexes the laser polarizations: left, linear and right.

In the following, we calculate the relativistic Rabi frequencies

2 $$\begin{aligned} \Omega _{(\lambda )}={\mathscr {A}}_{\mathbf {k}}\mid \hat{\varepsilon }_{\mathbf {k}}^{(\lambda )}\cdot {\varvec{\gamma }}_{21}\mid , \end{aligned}$$

where

3 $$\begin{aligned} {\varvec{\gamma }}_{21}=e\langle 2\mid {\varvec{\alpha }}\,e^{i{\mathbf {k}}\cdot {\mathbf {r}}}\mid 1\rangle , \end{aligned}$$

where ${\mathscr {A}}_{{\mathbf {k}}}=(1/2\omega _{L}V)^{1/2}$, $\hat{\varepsilon }_{{\mathbf {k}}}^{(\lambda )}$ is the polarization vector of the field, *e* the charge of the electron, ${\mathbf {k}}$ the wave number, ${\mathbf {r}}$ the position of the electron, and *V* the volume of the space in which we do the quantization. The $4\times 4$ Dirac matrices ${\varvec{\alpha }}\equiv (\alpha _1,\alpha _2,\alpha _3)$ have the representation^23^

4 $$\begin{aligned} \alpha _i=\begin{pmatrix} 0 &{}\quad \sigma _i \\ \sigma _i &{}\quad 0 \end{pmatrix}, \end{aligned}$$

where $\sigma _i$, $i\in \{1,2,3\}$, are the $2\times 2$ Pauli matrices. By definition

5 $$\begin{aligned} \hat{\varepsilon }_{{\mathbf {k}}}^{(\pm )}=\frac{1}{\sqrt{2}}({\hat{e}}_{x}\pm i{\hat{e}}_{y}), \end{aligned}$$

and describes left- and right-circularly polarized waves, with ${\hat{e}}_{x}$ and ${\hat{e}}_{y}$ the unit vectors along the *x* and *y*-axis, respectively. We consider the propagation vector ${\mathbf {k}}$ along the axis *z*, and consequently the polarization vector $\hat{\varepsilon }_{{\mathbf {k}}}^{(\pm )}$ is always perpendicular to ${\mathbf {k}}$. Dirac functions are obtained from the state vectors $\mid a\rangle$, $a=\overline{1,2}$, considering the inner product $\Psi _a(r)=\langle r\mid a\rangle$, whose representations we can find in^10^. We noted the index $a\equiv (n_a,j_a,l_a,M_a)$, where $n_a$ is the principal quantum number, $j_a$ and $l_a$ the angular momentum quantum numbers, and $M_a$ the magnetic quantum number.

To compute the matrix element (3), we must develop the exponential $e^{i{\mathbf {k}}\cdot {\mathbf { r}}}$ in spherical Bessel functions $j_l(kr)$, obtaining^24^

6 $$\begin{aligned} e^{i{\mathbf {k}}\cdot {\mathbf {r}}}=4\pi \sum _{l=0}^{\infty }\sum _{m=-l}^li^lj_l(kr)Y_{lm}^*({\hat{k}})Y_{lm}({\hat{r}}), \end{aligned}$$

where ${\hat{k}}={\mathbf {k}}/k$ and ${\hat{r}}={\mathbf {r}}/r$. The $Y_{lm}({\hat{r}})$ functions are known as Laplace’s spherical harmonics. Spherical Bessel functions can be evaluated using the formula

7 $$\begin{aligned} j_l(kr)= & {} \sqrt{\frac{\pi }{2kr}}\sum _{\beta =0}\frac{(-1)^\beta }{2^{2\beta +l+1/2}\beta !\Gamma (\beta +l+3/2)}(kr)^{2\beta +l+1/2}. \end{aligned}$$

**Figure 1:**
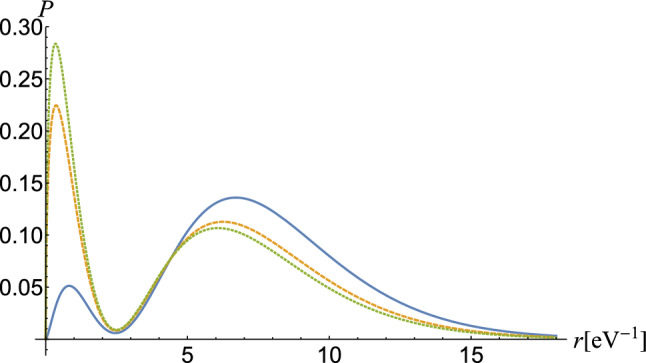
Probability density $P=r^2\mid \Psi _a^{{\mathrm {c}}}({\mathbf {r}})\mid ^2$ in a curved space of normalized wave functions for energy level 2*s* in the U$^{89+}$ ion. The case $b=0$ (continuous) corresponds to the flat space. As the *b* parameter increases, the probability density approaches the origin, meaning that the electron becomes more and more massive. The orange curve (dashed) corresponds to $b=5$ and the green one (dotted) to $b=10$.

The physics of relativistic quantum systems for 1/2 spin particles in Minkowski spaces is well known and is governed by the Dirac equation. In particular, the paper^25^ solved the Dirac equation using Melvin’s metric and the paper^15^ solved the Dirac equation in curved space-time in the presence of an electromagnetic field. The work^26^, studied analytical solutions for the Dirac equation with spherical symmetry in curved space-time, metric that we also use in this paper. It is defined8where *r* is the radial coordinate, $\theta$ and $\phi$ the angular coordinates, 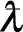 is the Compton wavelength, which in relativistic units is 1, and *b* is a dimensionless parameter. Eq. (8) has as special cases Schwarzschild^27^ and anti de-Sitter metrics^28^. In the case of curved space-time, the angular part of the line element is not changed from the usual Dirac equation, but the temporary component of the metric produces an extra curvature-dependent term. In this paper we will discuss how to determine the parameter *b*.

In order to obtain the spinor of the hydrogen atom in curved space-time, the constant mass term is modified with the position-dependent mass term^26^9The rest mass of the particle *m* is obtained either when $r\rightarrow \infty$ or when , i.e., at nonrelativistic limit. We must note that this position-dependent mass has a relativistic character, because it is canceled at the non-relativistic limit.

In conclusion, we can express the spinor $\Psi _a^{{\mathrm {c}}}({\mathbf {r}})$ of the hydrogen atom in curved space-time as a function of the spinor $\Psi _a({\mathbf {r}})$ of the hydrogen atom in flat space-time^26^, i.e.,

10 $$\begin{aligned} \Psi _a^{{\mathrm {c}}}({\mathbf {r}})=\left( 1+\frac{b}{r}\right) ^{1/2}\Psi _a({\mathbf {r}}). \end{aligned}$$

The relation is easy to understand in view of the fact that we normalize $\mid \Psi _a^{{\mathrm {c}}}({\mathbf {r}})\mid ^2=2m(r)c^2$, where *c* is speed of light in vacuum. If we note $\Psi _a^{{\mathrm {c}}}({\mathbf {r}})=\langle r\mid a\rangle ^c$, $a\in \{1,2\}$, in Eqs. (2) and (3), we need to replace $\mid a\rangle$ with $\mid a\rangle ^c$.

In the Fig. 1, we represented in natural units the probability $P=r^2\mid \Psi _a^{{\mathrm {c}}}({\mathbf {r}})\mid ^2$ of the level 2*s* of Li-like uranium, $Z=89$. The case $b=0$ (blue, continuous) corresponds to the flat space. The corresponding case $b=5$ is represented by orange (dashed) and the corresponding case $b=10$ by green (dotted). We notice that, with the increase of the parameter *b*, the peak of the figure approaches the origin, which means that the electron becomes more and more massive.

By calculations analogous to those performed in the work^10^, we can find the following results

11 $$\begin{aligned} \hat{\varepsilon }_{{\mathbf {k}}}^{(+)}\cdot {\varvec{\gamma }}_{21}^c= & {} e\sqrt{4\pi }\sum _li^{l}\sqrt{2l+1}{}^c\langle 1\mid \hat{\varepsilon }_{\mathbf {k}}^{(+)}\cdot {\varvec{\alpha }}\, j_l(kr)Y_{l0}(\hat{r})\mid 2\rangle ^c\nonumber \\= & {} -e\sqrt{2\pi }\sum _li^{l-1}\sqrt{2l+1}\left\{ -R_1^l[K_{1x}^l+iK_{1y}^l]+R_2^l[K_{2x}^l+iK_{2y}^l]\right\} , \end{aligned}$$

and

12 $$\begin{aligned} \hat{\varepsilon }_{\mathbf {k}}^{(-)}\cdot {\varvec{\gamma }}_{21}^c= & {} e\sqrt{4\pi } \sum _li^{l}\sqrt{2l+1}{}^c\langle 1\mid \hat{\varepsilon }_{\mathbf {k}}^{(-)}\cdot {\varvec{\alpha }}\,j_l(kr)Y_{l0}({\hat{r}})\mid 2\rangle ^c \nonumber \\= & {} -e\sqrt{2\pi }\sum _li^{l-1}\sqrt{2l+1}\left\{ -R_1^l\left[ K_{1x}^l-iK_{1y}^l\right] +R_2^l\left[ K_{2x}^l-iK_{2y}^l\right] \right\} . \end{aligned}$$

Using Eqs. (11) and (12), we obtain

13 $$\begin{aligned} \hat{\varepsilon }_{\mathbf {k}}^{(0)}\cdot {\varvec{\gamma }}_{21}^c=\frac{1}{\sqrt{2}}\left( \hat{\varepsilon }_ {\mathbf {k}}^{(+)}\cdot {\varvec{\gamma }}_{21}^c+\hat{\varepsilon }_{\mathbf {k}}^{(-)}\cdot {\varvec{\gamma }}_{21}^c\right) =e\sqrt{4\pi }\sum _li^{l-1}\sqrt{2l+1}\left\{ R_1^lK_{1x}^l-R_2^lK_{2x}^l\right\} . \end{aligned}$$

**Figure 2:**
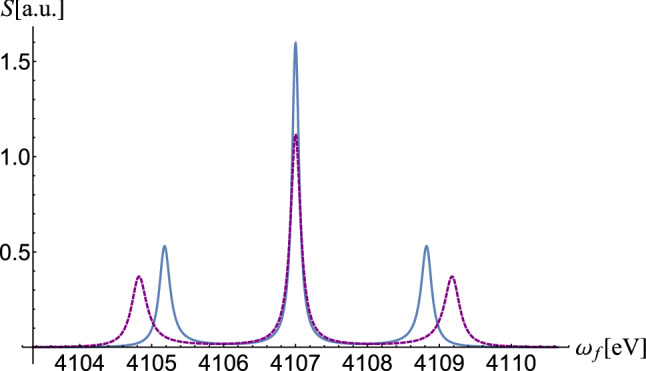
Fluorescence spectrum for $2s\leftrightarrow 2p_{3/2}$ transition in Li-like uranium as a function of fluorescence photon frequency $\omega _f$ at resonance $\omega _L=\omega _{12}=$ 4106.6 eV. The laser intensity is $10^{14}$ W/cm${}^{2}$. With blue (continuous) we represented the case $b=0$, and with purple (dashed) the case $b=5$.

The angular part is identical to the angular part described for the flat space^10^, i.e.,

14 $$\begin{aligned} K_{1x}^l= & {} \int do_r \Omega _{-\kappa _1M_1}^{\dagger }({\hat{r}})\sigma _{x}Y_{l0}\Omega _{\kappa _2M_2}({\hat{r}}),\nonumber \\ K_{2x}^l= & {} \int do_r \Omega _{\kappa _1M_1}^{\dagger }({\hat{r}})\sigma _{x}Y_{l0}\Omega _{-\kappa _2M_2}({\hat{r}}),\nonumber \\ K_{1y}^l= & {} \int do_r \Omega _{-\kappa _1M_1}^{\dagger }({\hat{r}})\sigma _{y}Y_{l0}\Omega _{\kappa _2M_2}({\hat{r}}),\nonumber \\ K_{2y}^l= & {} \int do_r \Omega _{\kappa _1M_1}^{\dagger }({\hat{r}})\sigma _{y}Y_{l0}\Omega _{-\kappa _2M_2}({\hat{r}}), \end{aligned}$$

where $\Omega _{\kappa _aM_a}$ is the spherical spinor, and where the Pauli $2\times 2$ matrices ${\varvec{\sigma} }$ are defined via ${\varvec{\sigma }}=\sigma _{x}{\hat{e}}_{x}+\sigma _{y}{\hat{e}}_{y}+\sigma _{z}{\hat{e}}_{z}$. The result of these integrals is presented in^10^.

The radial part consists of integrals

15 $$\begin{aligned}&R_1^l=\int _0^{\infty } dr r^2 m(r) F_{n_1\kappa _1}(r)j_l(kr)G_{n_2\kappa _2}(r),\nonumber \\&R_2^l=\int _0^{\infty } dr r^2 m(r) G_{n_1\kappa _1}(r)j_l(kr)F_{n_2\kappa _2}(r), \end{aligned}$$

where the radial functions $G_{n_a\kappa _a}$ and $F_{n_a\kappa _a}$, $a\in \overline{1,2}$, can be expressed as mathematical analytic functions and are given in^10^. The result of the radial integration is presented in the Appendix.

To calculate the decay widths $\Gamma$, we use the theory presented in^29^, in which we operate the modification (10). Because ${\varvec{\gamma }}_{21}^c\sim$ $\hat{\varepsilon }_{\mathbf {k}}^{(\lambda )}\cdot$ $^c\langle 2\mid {\varvec{\alpha }}\,e^{i{\mathbf {k}}\cdot {\mathbf {r}}}\mid 1\rangle ^c$ $=C\,\hat{\varepsilon }_{\mathbf {k}}^{(\lambda )}\cdot \langle 2\mid {\varvec{\alpha }}\,e^{i{\mathbf {k}}\cdot {\mathbf {r}}}\mid 1\rangle$, where *C* is a constant that is determined directly from the calculation, and $\Gamma ^c\sim \mid \hat{\varepsilon }_{\mathbf {k}}^{(\lambda )} \cdot ^c\langle 2\mid {\varvec{\alpha }}\,e^{i{\mathbf {k}}\cdot {\mathbf {r}}}\mid 1\rangle ^c\mid ^2$, in the graphical representation of the fluorescence spectrum for electrical transitions we can estimate $\Gamma ^c\approx C^2\,\Gamma$.

**Figure 3:**
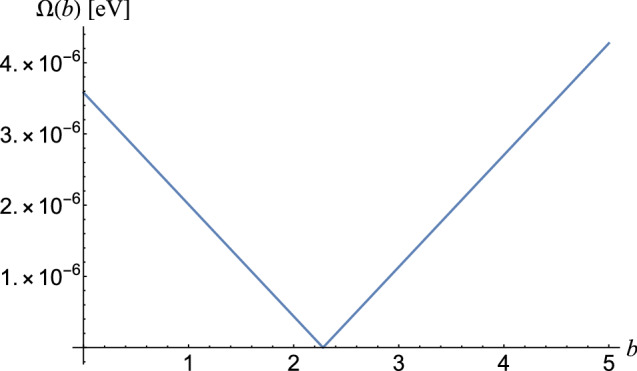
Dependence of Rabi frequency on the parameter *b* in U$^{90+}$, for the transition $2s\rightarrow 2p_{3/2}$ for the intensity $I=10^{14}$ W/cm$^2$.

## Results

On the one hand, according to Einstein’s famous energy-mass relationship, energy turns into mass and mass turns into energy. On the other hand, according to general relativity, mass is responsible for curvature, and with increasing mass there is an increase in curvature. High power lasers offer high energy, and consequently we expect the space to bend. The *b* parameter in Eq. (10) is the representative of the curvature in the metric, and is the one that creates the correspondence between the intensity of the laser and the metric. Note that *b* is actually *b*(*I*), where *I* is the intensity of the laser, but to simplify the notation we call it only *b*.

Rabi frequencies are dependent on laser polarization, and in ascending order, we distinguish the following situations: polarized left, linear and right. To determine the parameter *b* we can consider any of the polarizations mentioned above. In this paper we will consider the laser polarized on the right, because it corresponds to the highest Rabi frequency.

To reduce the ionization process for the interaction of high-power lasers with few-electron ions, we must use high-*Z* ions. Therefore, in order to be able to make the measurements suggested in this paper, we need highly charged ions, which can only be described by relativistic theory: spinors. At high-*Z*, the electron-electron correlation can be neglected, and we can use as wave functions the solutions of the Dirac equations for the hydrogen atom^30^, in which *Z* is replaced with effective *Z*. In^31^, the authors calculated relativistic theory of internal bremsstrahlung in electron capture, concluding that the alteration in the structure of the propagator caused by screening, is negligible. Further, considering the lack of correlation in spectral photon distribution from the $1s2s^1S_0\rightarrow 1s^2$ ${}^1S_0$ two-photon decay, the authors obtained excellent agreement between experiment and theory^32^. Last but not least, we would like to mention the work^33^, in which analyzing the two-photon decay process of $2\,{}^1S_0$ and of $2\,{}^3S_1$, the authors concluded that the correlation effects become less important with increasing nuclear charge.

A Li-like ion is an ion with the fundamental level 1*s* occupied and an electron on the level 2*s*. In flat space, the energy between the level 2*s* and the excited level $2p_{3/2}$ is in the X-ray range. Because U$^{90+}$ has strong Coulomb potential, the electron flopping between the energy levels 2*s* and $2p_{3/2}$ is difficult to ionize even in high intensity laser fields.

In U$^{90+}$ the width of the central line is $\Gamma =0.12$ eV. For an intensity of $10^{14}$ W/cm$^2$, the Rabi frequency of 1.82 eV exceeds the width of the central line giving rise to a Mollow spectrum with three peacks. In Fig. 2, we represented in blue (continuous) the resonant power spectrum for flat space ($b=0$), and in purple (dashed) we represented the one corresponding to the curved space for $b=5$. It is clear that the two spectra are distinct. The central spectra of the two figures are centered in the same energy, although the energy depends on the parameter *b* according to Eq. (19). In fact, we are interested in the distance between the sidebands.

To establish the correlation between the parameter *b* and the laser intensity *I*, we use the following procedure: we first use the low-intensity Mollow spectrum to determine the Rabi frequency, which has an intensity dependence of the form $\sim \sqrt{I}$. For high laser intensities we expect the Rabi frequency to not satisfy this relationship. On the other hand, in Fig. 3, we represented the Rabi frequency in a curved space as a function of the parameter *b*, and we find that we obtain a linear dependence. On this simple graph, we read the parameter *b* corresponding to the measured Rabi frequency (which will not correspond to $b=0$). Thus, the correspondence between the parameter *b* and the intensity *I* can be determined experimentally.

*m*(*r*) from Eq. (9) creates in Eqs. (15) two distinct radial integrals of different signs, one of electric type corresponding to the constant *m* and one of magnetic type for
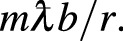
. The parameter *b* satisfies an equation of form

16 $$\begin{aligned} \Omega (b)=\mid -\Omega +b\,{\mathscr {C}}\Omega '\mid \sqrt{I}, \end{aligned}$$

where the values $\Omega$ and $\Omega '$ are given in Table 1, *I* is given in W/cm$^2$, and ${\mathscr {C}}=1$ W$^{-1}$, is a constant that establishes the dimensionality. The value of $\Omega$ corresponds to the case when $b=0$.

As a consequence of Eq. (1), the maximum signal is obtained at the resonance. We wonder how they are affected by measurement if we are not at resonance. The distance between the sidebars is given by the formula

17 $$\begin{aligned} D(\Delta )=2\Omega _{(\lambda )}+\Omega _{(\lambda )}\left( \frac{\Delta }{\Omega _{(\lambda )}}\right) ^2 +{\mathscr {O}}\left( \left( \frac{\Delta }{\Omega _{(\lambda )}}\right) ^4\right) . \end{aligned}$$

As the Rabi frequency depends directly proportional to the square root of the intensity, the dependence of the distance between the detuning bands becomes weaker as the laser intensity increases. And we want as much intensity as possible, because we expect the scalar curvature to increase with increasing intensity. The relative accuracy defined as $\Gamma _{SB}/\Omega _{(\lambda )}$, with $\Gamma _{SB}$ the width of the side bands, also increases with laser intensity.

**Figure 4:**
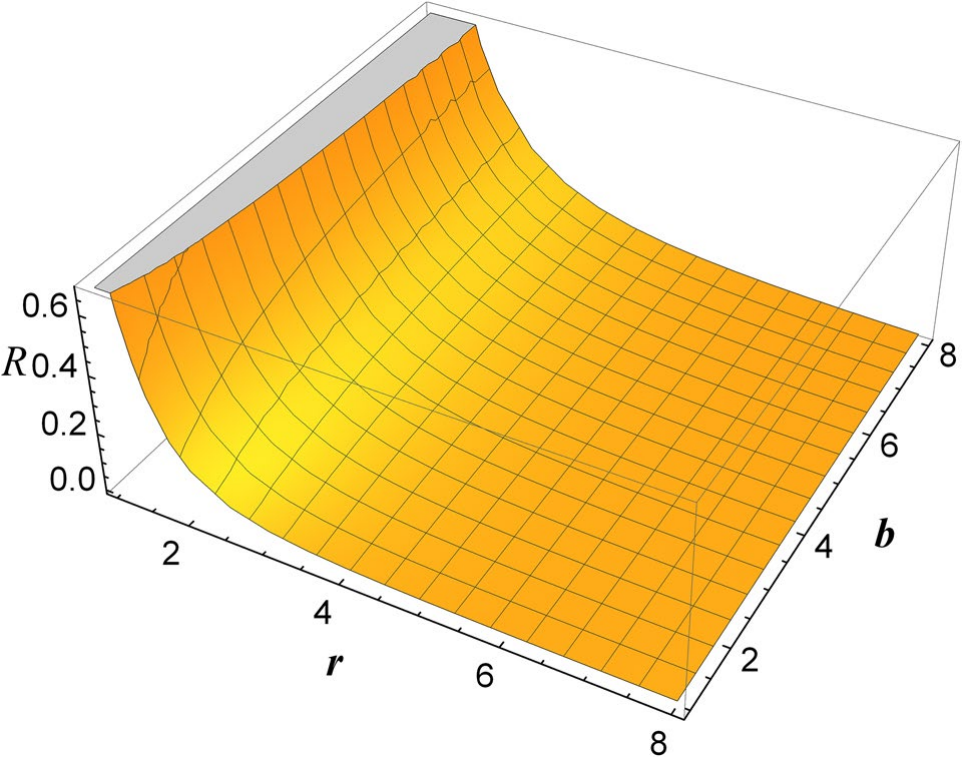
Scalar curvature *R*, for $b\in [1,8]$, and $r\in [1,8]$ Bohr radii.

Considering in Eq. (7) $n=0$, we obtain the results from the dipole approximation. In this paper we add up to $n=3$, which leads to the change of Rabi frequencies, ensuring the correctness of the result for the first three digits after the comma. This effect is called retardation (see e.g.^29^). Because the second contribution is negative, the retardation produces lower Rabi frequencies than in the dipole approximation.

Using Fig. 2 together with Fig. 3, we can determine the parameter *b* necessary to determine the scalar curvature^27^

18 $$\begin{aligned} R=\frac{2 b^2}{(r+ b)^4}-\frac{4b}{(r+b)^3}-\frac{2}{(r+b)^2}+\frac{2}{r^2}. \end{aligned}$$

In Fig. 4, we represented the scalar curvature *R*, for $b\in [1,8]$, and $r\in [1,8]$ Bohr radii. The small ripples in the grid of the figure must be ignored, because they represent numerical errors when *b* is large and *r* is small.

In the Minkowsky space, the energies of the electrons bound in the Coulomb field, with position-independent mass, are given by Sommerfeld’s formula. In curved space-time, the effective mass of a spinor described by Eq. (9) has a relativistic component which is proportional to 1/*r*, and consequently, the Hamiltonian of the system contains this additional term, complicating the calculations. It should be noted that the rest mass of the particle ($m_0=m$) is obtained when , which is actually the non-relativistic limit. This connection between relativistic and non-relativistic behavior allows the authors of^34^ to establish the following formula for the relativistic spectrum in curved space-time

$$\begin{aligned} E_{al}=\left( 1+\left( \frac{Z}{a+l_a'+1}\right) ^2\right) ^{-1}\left( -\frac{bZ}{(a+l_a'+1)^2}\pm \sqrt{1+\frac{Z^2-b^2}{(a+l_a'+1)^2}}\right) , \end{aligned}$$

with $\gamma _a=\frac{\mid \kappa _a\mid }{\kappa _a}\sqrt{\kappa _a^2+b^2-Z^2}$, where $\kappa _a$ is the spin-orbit quantum number defined as $\kappa _a=\pm (j_a+1/2)$ for $l_a=j_a\pm 1/2$. In the above equation, for $\kappa >0$ we have $l_a'=\gamma _a$ and for $\kappa <0$, $l_a'=-\gamma _a-1$. In the case of flat space ($b=0$), the known Sommerfeld formula is obtained. Using Figs. 2 and 3, we can determine the parameter *b* and consequently establish the atomic spectrum.

Employing the present laser facilities^35,36^, our method is limited due to the short pulse duration of the laser (of $\sim 300$ fs) and its dephasing width $\gamma _D$, typically on the order of 1 eV. The widths of the sidebands $\Gamma _{SB}$ have the expression^10^ $\Gamma _{SB}=(3\Gamma +\gamma _D)/4$, therefore, the widths are roughly a quarter of dephasing. Until the laser dephasing improves, one may extend the present theory to three-level systems driven by an X-ray ($1\rightarrow 3$) and an optical laser-field ($1\rightarrow 2$)^11^. The outer sidebands are given by $\Gamma _{SB}=\big | \frac{3}{2}(\Gamma _{31}-\frac{1}{3}\gamma _D)R + \frac{1}{2}\Gamma _{32}(R+R^2) + \frac{3}{2}\Gamma _{21}(1-R) \big |$ with the ratio *R* being $\Omega _{31}^2/(\Omega _{31}^2+\Omega _{21}^2)$. Increasing the intensity in the $2\rightarrow 1$ transition, we get $R\rightarrow 0$, and $\Gamma _{SB}\rightarrow \frac{3}{2}\Gamma _{21}$, so we can get $\Gamma _{SB }$ independent of $\gamma _D$.

**Table 1:** Parameters for the $2s\leftrightarrow 2p_{3/2}$ E$_1$ transitions in Li-like ions. Transition energies $\omega _{12}$, natural line width $\Gamma$, and Rabi frequencies $\Omega$ are given for the laser intensities $I=10^{14}$ W/cm$^2$. *x*(*y*) stands for $x\times 10^{y}$.

	$\omega _{12}$ [eV]	$\Gamma$ [eV]	$\Omega$ [eV]	$\Omega '$ [eV]
Kr	6.294(1)	5.931(-6)	5.262	9.019
Xe	3.641(2)	4.761(-4)	3.388	3.555
Nd	5.776(2)	1.508(-3)	3.016	2.752
U	4.107(3)	1.200(-1)	1.829	0.802

## Conclusions and outlook

The energy-level shifts of hydrogen in the space curved by the intense short laser pulses in the nonrelativistic limit, are studied in^37^. It was shown in^38^ that the energy levels of the atoms in the curved spacetime would be displaced due to the local spacetime curvature. The energies of the different levels are modified differently by the laser intensity and, consequently, the curvature effect can be distinguished from other effects.

In this paper, using the relativistic theory of resonance fluorescence in a curved space-time, we can determine the scalar curvature produced by a high-power laser in a relativistic ion. Due to the fact that in heavy ions electrons are strongly bound to the nucleus by Coulomb force, they oppose ionization and allow the study of population oscillation between two different energy levels. The model can be extended to a system with three energy levels driven by two laser fields where the bandwidth of the spectrum can be considerably reduced by several orders of magnitude due to the interference effect, allowing a more precise determination of the scalar curvature.

It is important to have a measuring instrument that can test regions with high curvature at great distances from us. The atom through its spectrum provides such an instrument. This article also provides a way to see if Einstein’s gravitational theory is valid in the dimension of laboratory.

In this paper we have adopted the Mollow’s work, where it is assumed that the atom is driven by a perfectly monochromatic field. The present work can be extended to the case of a pulse-train driving field. In this case, the calculation technique must be adapted to the response of a high-resolution Fabry–Perot interferometer to a narrow-band-width classical input field with a periodic envelope^39^, and consequently the observable power spectrum must be averaged over time.

## Supplementary Information

Supplementary Information.

## Data Availability

All data generated or analysed during this study are included in this published article and its supplementary information files.
