# Supplementary material for: Measurement of the scalar curvature of high-power lasers
Source: Sci Rep. 2022 Oct 27;12:18057. doi: 10.1038/s41598-022-23045-8 (PMC9613682; doi:10.1038/s41598-022-23045-8)
Supplement: Supplementary file 1 — Supplementary Information. [file 41598_2022_23045_MOESM1_ESM.pdf]

Measurement of the scalar curvature of high-power lasers

Antonela Toma[1] **and Octavian Postavaru**[1,*]

[1]Center for Research and Training in Innovative Techniques of Applied Mathematics in Engineering, University Politehnica of Bucharest, Splaiul Independentei 313, Bucharest, 060042, Romania
[*]opostavaru@linuxmail.org

Appendix

In this section, we present the calculation of the radial part. After integrating Eqs. (15), it is obtained

$$\begin{aligned} R_1^l &= -\sqrt{\pi}(1-E_1)^{1/2}(1+E_2)^{1/2}U_1U_2\sum_{\beta=0}^{\infty}\frac{(-1)^{\beta}k^{2\beta+l}}{2^{2\beta+l+1}\beta!\Gamma(\beta+l+3/2)}\left(R_{11}^{l\beta}-R_{12}^{l\beta}+R_{13}^{l\beta}-R_{14}^{l\beta}\right), \qquad (1)\\ R_2^l &= -\sqrt{\pi}(1+E_1)^{1/2}(1-E_2)^{1/2}U_1U_2\sum_{\beta=0}^{\infty}\frac{(-1)^{\beta}k^{2\beta+l}}{2^{2\beta+l+1}\beta!\Gamma(\beta+l+3/2)}\left(R_{11}^{l\beta}+R_{12}^{l\beta}-R_{13}^{l\beta}-R_{14}^{l\beta}\right), \end{aligned}$$

where $R_{1i}^{l\beta}=R_{1i}^{l\beta 1}+bR_{1i}^{l\beta 0}$, $i=\overline{1,4}$, with

$$\begin{aligned} R_{11}^{l\beta\delta} &= n_1^r n_2^r\sum_{m=0}^{n_1^r-1}\frac{(-n_1^r+1)_m}{(2\gamma_1+1)_m}H^{l\beta\delta m}{}_2F_1\left(x^{l\beta\delta m},-n_2^r+1,2\gamma_2+1;\frac{2\lambda_2}{\lambda_1+\lambda_2}\right), \qquad (2)\\ R_{12}^{l\beta\delta} &= n_1^r(N_2-\kappa_2)\sum_{m=0}^{n_1^r-1}\frac{(-n_1^r+1)_m}{(2\gamma_1+1)_m}H^{l\beta\delta m}{}_2F_1\left(x^{l\beta\delta m},-n_2^r,2\gamma_2+1;\frac{2\lambda_2}{\lambda_1+\lambda_2}\right),\\ R_{13}^{l\beta\delta} &= (N_1-\kappa_1)n_2^r\sum_{m=0}^{n_1^r}\frac{(-n_1^r)_m}{(2\gamma_1+1)_m}H^{l\beta\delta m}{}_2F_1\left(x^{l\beta\delta m},-n_2^r+1,2\gamma_2+1;\frac{2\lambda_2}{\lambda_1+\lambda_2}\right),\\ R_{14}^{l\beta\delta} &= (N_1-\kappa_1)(N_2-\kappa_2)\sum_{m=0}^{n_1^r}\frac{(-n_1^r)_m}{(2\gamma_1+1)_m}H^{l\beta\delta m}{}_2F_1\left(x^{l\beta\delta m},-n_2^r,2\gamma_2+1;\frac{2\lambda_2}{\lambda_1+\lambda_2}\right), \end{aligned}$$

and with

$$\begin{aligned} H^{l\beta\delta m} &= (2\lambda_1)^{\gamma_1-1}(2\lambda_2)^{\gamma_2-1}\frac{(2\lambda_1)^m}{m!}\frac{\Gamma(m+\gamma_1+\gamma_2+\delta+2\beta+l)}{(\lambda_1+\lambda_2)^{m+\gamma_1+\gamma_2+\delta+2\beta+l}},\\ x^{l\beta\delta m} &= m+\gamma_1+\gamma_2+\delta+2\beta+l. \end{aligned}$$

In the above equations, we used the following notations

$$\begin{aligned} \lambda_a &= \sqrt{1-E_a^2}, \quad N_a=\frac{n_a^r+\gamma_a}{E_a}, \qquad (3)\\ \gamma_a &= \sqrt{\kappa_a^2-(Z\alpha)^2}, \quad n_a^r=a-|\kappa_a|,\\ \kappa_a &= \begin{cases} -(l_a+1) & \text{if } j_a=l_a+\frac{1}{2},\\ l_a & \text{if } j_a=l_a-\frac{1}{2}, \end{cases} \end{aligned}$$

where $a=\overline{1,2}$.
